# The Toll-Like Receptor 3 Agonist Polyriboinosinic Polyribocytidylic Acid Increases the Numbers of NK Cells with Distinct Phenotype in the Liver of B6 Mice

**DOI:** 10.1155/2020/2489407

**Published:** 2020-03-05

**Authors:** Mohamed L. Salem, Sabry A. El-Naggar, Maysa A. Mobasher, Rehab M. Elgharabawy

**Affiliations:** ^1^Immunology and Biotechnology Unit, Zoology Department, Faculty of Science, Tanta University, Tanta, Egypt; ^2^Center of Excellence in Cancer Research, New Tanta University Teaching Hospital, Tanta University, Egypt; ^3^Biochemistry Division, Department of Pathology, College of Medicine, Jouf University, Sakakah, Saudi Arabia; ^4^Department of Clinical Pathology, El Ahrar Educational Hospital, Ministry of Health, Zagazig, Egypt; ^5^Department of Pharmacology and Toxicology, College of Pharmacy, Qassim University, Buraydah, Saudi Arabia; ^6^Department of Pharmacology and Toxicology, Faculty of Pharmacy, Tanta University, Tanta, Egypt

## Abstract

One of the activating factors of the cells of the innate immune system is the agonists of toll-like receptors (TLRs). Our earlier publications detailed how poly(I:C), a TLR3 agonist, elevates the NK cell population and the associated antigen-specific CD8^+^ T cell responses. This study involved a single treatment of the B6 mice with poly(I:C) intraperitoneally. To perform a detailed phenotypic analysis, mononuclear cells were prepared from each of the liver, peripheral blood, and spleen. These cells were then examined for their NK cell population by flow cytometric analysis following cell staining with indicated antibodies. The findings of the study showed that the NK cell population of the liver with an NK1.1^high^CD11b^high^CD11c^high^ B220^+^Ly6G^−^ phenotype was elevated following the treatment with poly(I:C). In the absence of CD11b molecule (CR3^−/−^ mice), poly(I:C) can still increase the remained numbers of NK cells with NK1.1^+^CD11b^−^ and NK1.1^+^Ly6G^−^ phenotypes in the liver while their numbers in the blood decrease. After the treatment with anti-AGM1 Ab, which induced depletion of NK1.1^+^CD11b^+^ cells and partial depletion of CD3^+^NK1.1^+^ and NK1.1^+^CD11b^−^ cell populations, poly(I:C) normalized the partial decreases in the numbers of NK cells concomitant with increased numbers of NK1.1^−^CD11b^+^ cell population in both liver and blood. Regarding mice with a TLR3^−/−^ phenotype, their injection with poly(I:C) resulted in the partial elevation in the NK cell population as compared to wild-type B6 mice. To summarise, the TLR3 agonist poly(I:C) results in the elevation of a subset of liver NK cells expressing the two myeloid markers CD11c and CD11b. The effect of poly(I:C) on NK cells is partially dependent on TLR3 and independent of the presence of CD11b.

## 1. Introduction

Natural killer (NK) cells are immune cells that target and kill cells infected with viruses or tumourigenic cells [1–3]. They are able to differentiate between such cells and normal cells, and therefore spare normal cells [1–3]. Other characteristics that are also attributed to NK cells include the release of cytokines and chemokines that are vital for the instigation and amplification of an inflammatory response [4–6]. Such chemokines and cytokines include TNF-*α*, IFN-*γ*, IL-10, GM-CSF, and IL-13 [4–6]. Such functions of NK cells may help understand the essential role played by these cells in conducting immune surveillance against tumours and in the eradication of circulating cancer cells in mice [2, 5, 7].

Previous studies have also indicated that cancer cells in the liver or those that metastasise to the lungs can be killed and eliminated from the liver by a specific subset of liver NK cells [8–10]. NK cells are capable of secreting inflammatory cytokines that can result in Th1 cell activation, and it is this ability that is reported to be responsible for their antitumour activity [11, 12]. Specifically, this action of NK cells tends to be instigated following binding of the toll-like receptors (TLRs) they express to its specific ligands [11, 12]. Furthermore, as specific subsets of the NK cell population may convey a regulatory action [13–15], it is important to explore the specific NK cell subset that is influenced by the TLR agonists.

Following the TLR's linking with particular ligands, the array of cytokines, chemokines, and other proinflammatory mediators that are released leads to the communication between innate immunity and adaptive immunity, ultimately resulting in dendritic cell (DC) maturation [16–20]. The joint action of the mature DCs together with the proinflammatory mediators has a direct impact on NK cells leading to their activation and proliferation, the initiation of their cytotoxic functions [18, 21], all of which led to stimulation of cytotoxic T lymphocytes [22, 23]. TLRs are able to identify specific microbe-associated ligands, and in a number of cells of the innate immunity specifically in NK and DC cells and macrophages, these TLRs are present in large numbers [16, 24–26]. TLR3 is capable of identifying double-stranded RNA (dsRNA) which is produced through the replication of viruses. TLR3 is also capable of recognising poly(I:C), polyriboinosinic polyribocytidylic acid, which is the synthetic form of drRNA [27–29]. Poly(I:C) is known to initiate antitumour actions due to its activation of both NK and DC cell populations [18, 21, 26, 30]. Regarding this action, our earlier publications detailed how the treatment with poly(I:C) results in the instigation of early (1 hr following injection) rise in the NK cells of the liver, specifically those with an NK1.1^+^CD11b^+^ phenotype. Four hours following this induction, a subsequent rise is also seen in cells with an NK1.1^+^CD11c^+^ phenotype and DC cells with a CD11c^+^CD11b^+^ phenotype [18]. The impact of poly(I:C) on NK cells was linked to its capability to release inflammatory cytokines, which was coincided with its adjuvant impact on CD8^+^ T cells that are specific to OVA albumin MHC class-I peptide SIINFEKL vaccination [18]. Moreover, our studies together with others have shown the adjuvant impact of poly(I:C) on the antigen-specific CD8^+^ T cell responses in part are mediated by NK cells through its release of inflammatory cytokines, particularly including MCP-1, IL-6, and TMF-*α* formation of a fast cytokine milieu [18, 21, 30–35]. The poly(I:C) treatment has also been shown to preferentially initiate the stimulation of liver NK cells [36]. Moreover, their migration to the spleen was associated with increased expression of cytokines [37]. These studies may explain the reported antitumour impact of poly(I:C) on tumour metastatic models [10, 38]. The in vitro studies showed the direct impact of poly(I:C) on NK cells, where the exposure of purified NK cells to poly(I:C) led to the rise in their expression of CD69, an activation marker, and also of their cytotoxicity [25, 30, 34].

Given the abovementioned reports on the important role of NK cells in linking innate and adaptive immunity and the stimulation of these cells by poly(I:C), this study is aimed at conducting an in-depth phenotypic analysis of NK cell populations immediately following the treatment with poly(I:C). This would help establish the exact NK cell phenotype that is stimulated by poly(I:C), a TLR3 agonist. The findings indicated that poly(I:C) preferentially results in a rise in hepatic NK cells with an NK1.1^+^CD11b^+^CD11c^+^B220^+^ Ly6G^−^ phenotype. These findings may understand the clinical employment of poly(I:C) and the role played by NK cells in antitumour and antimicrobial immunity.

## 2. Materials and Methods

### 2.1. Poly(I:C) Treatment

Two forms of B6 mice, C57BL/6 wild-type B6 mice (females) and CR3^−/−^ and TLR3^−/−^ B6 mice, were treated intraperitoneally with 200 *μ*g of poly(I:C) bought from InvivoGen (San Diego, CA). Poly(I:C) was dissolved in 200 *μ*l PBS prior to injection. One injection of 50 *μ*l of anti-asialo GM1 (AGM1) polyclonal antibody (Sigma-Aldrich, San Diego, CA) was given to the wild-type B6 mice to deplete them of NK cells. This was injected between 6 hr and 12 hr prior to their injection with poly(I:C). Flow cytometry was employed to confirm NK cell depletion by staining with anti-NK 1.1 mAb (Pharmingen, San Diego, CA, USA).

### 2.2. Sample Preparation

The mice were bled at four time points: 1 hr, 4 hr, 8 hr, and 12 hr following the poly(I:C) treatment. The peripheral blood leukocytes were prepared from the blood, and the mice were euthanised to harvest the spleen and the liver. A single-cell suspension was formed of both organs. ACK buffer (BD) was used to lyse the cells, followed by use of Ficoll Histopaque (Sigma-Aldrich, USA) for leukocyte purification. Ficoll was also used to prepare the cells for processing through flow cytometry. Next, the cell monolayer was centrifuged and then separated, and the full count of mononuclear cells per compartment was conducted using a haemocytometer. Finally, cell viability was examined using the trypan blue exclusion assay.

### 2.3. Flow Cytometry

As previously detailed [39], flow cytometry was employed for the measurement of cell surface analysis. First, anti-CD16/CD32 was used to treat fresh cells (1 × 10^6^) for 5 minutes. This was conducted on ice in a dark room. Conjugated FITC-, PE-, APC-, and cychrome-conjugated mAbs were then used to stain the cells. All monoclonal antibodies (mAbs) were bought from Pharmingen (San Diego, CA). This included anti-CD16/CD32, anti-NK1.1, anti-CD11b, anti-CD11c, anti-CD25, anti-B220, anti-Ly6G, anti-CD3, anti-CD40, and anti-CD80 mAbs. Following addition of the mAbs, the cells were incubated on ice in the dark for 30 minutes. The cells were then washed twice followed by their resuspension in 0.3 ml of 0.5% BSA, 0.02% sodium azide solution. A FACSCalibur flow cytometer was then employed for quantification of cell surface immunofluorescence. CellQuest software was then used to analyze the data. Each sample generated at least 50,000 events. Furthermore, at each time point, each compartment's complete cell population was counted. The five forms of cells derived from the flow cytometric analysis were used to determine the absolute number of cells by multiplying these five into the complete number of mononuclear cells derived from the peripheral blood.

### 2.4. Statistics

To conduct the statistical analysis, Student's *t*-test was used as the most suitable methodology, and this was conducted using two-sided *P* values, with *P* ≤ 0.05 indicating as significant.

## 3. Results

### 3.1. The Transient Reduction of NK1.1^+^CD11b^+^ Cells in the Peripheral Blood Is Linked to the Rise in Their Numbers in the Liver

**Figure 1:**
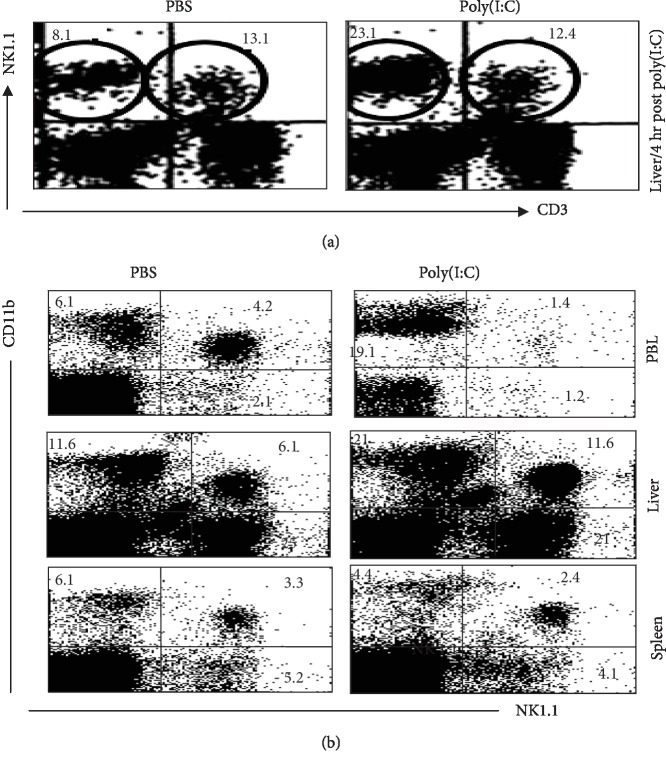
Treatment with poly(I:C) induces increased relative numbers of NK cells in the liver but decrease in the blood. Naïve C57BL/6 mice (*n* = 6 mice/group) were treated with 200 *μ*g poly(I:C) in 200 *μ*l and then sacrificed 4 hr post poly(I:C) injection. Single-cell suspensions were prepared of PBL and liver and stained with antibodies against NK1.1, CD3, and CD11b. Samples were analyzed by 3-color flow cytometry. The frequency of NK cells (NK1.1^+^CD3^−^) and NKT cells (NK1.1^+^CD3^+^) is shown in (a). The frequencies of CD11b^+^NK1.1^−^ and CD11b^+^NK1.1^+^ cells are shown in (b).

**Figure 2:**
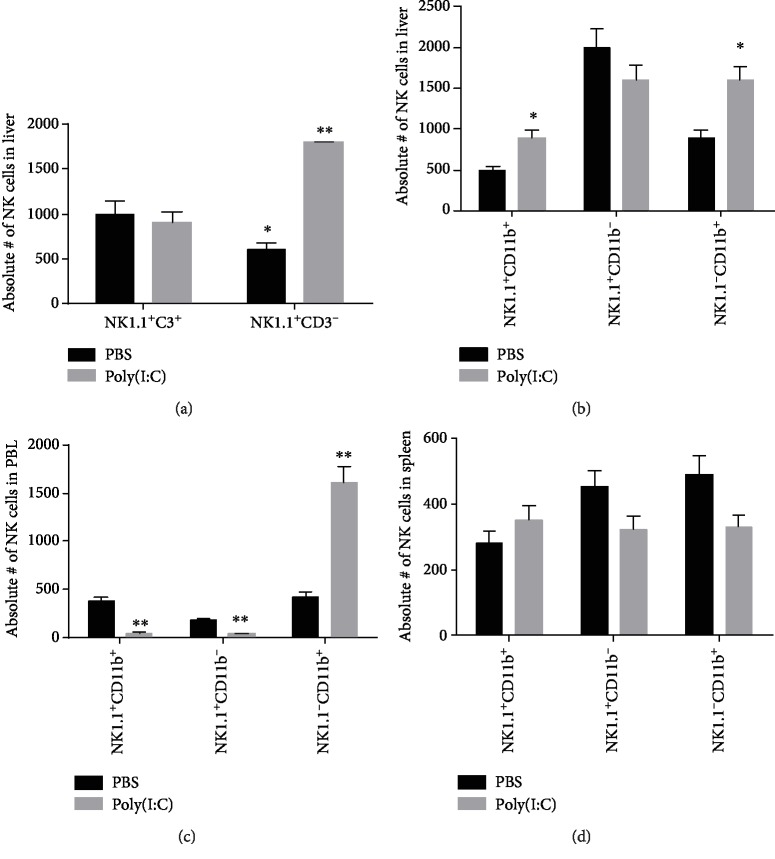
Treatment with poly(I:C) induces increased absolute numbers of NK cells in the liver but decrease in the blood. The absolute numbers of NK cells (NK1.1^+^CD3^−^) and NKT cells (NK1.1^+^CD3^+^) as well as CD11b^+^NK1.1^−^ and CD11b^+^NK1.1^+^ cells were calculated by multiplying the relative numbers analyzed in the legend of Figure 1 by the total numbers of the peripheral blood and liver by a haemocytometer. The data represents the mean ± SD of each group. ^∗^*P* < 0.05, ^∗∗^*P* < 0.01.

Phenotypically, NK cells are split into two cell populations, namely, NK cells that express the NK1.1^+^CD3^−^ phenotype and NKT cells that express the NK1.1^+^CD3^+^ phenotype. The former type of NK cells can be further split into the CD11b^−^ and the CD11b^+^ subsets. In this study, we noted that 4 hr following the treatment with poly(I:C), the liver had an increase in the relative numbers of NK1.1^+^CD3^−^ cells, but not NK1.1^+^CD3^+^ cells as illustrated in Figure 1(a). The numbers of these cell populations, however, did not change in the spleen (data not presented). The numbers of NK1.1^+^CD11b^+^ cells were reduced in the peripheral blood leukocytes (PBL) (the upper panel of Figure 1(b)) and elevated in the liver (the middle panel of Figure 1(b)). The poly(I:C) treatment, however, did not impact the numbers of NK1.1^+^CD11b^−^ cells in any of the spleen, liver, or PBL, despite raising numbers of NK1.1^−^CD11b^+^ cells in the liver and PBL, but not the spleen (Figure 1(b)). Of note, the changes in the relative number of these NK cell subsets in the liver following the treatment with poly(I:C) were in keeping with their absolute numbers (Figures 2(a)– 2(d)).

**Figure 3:**
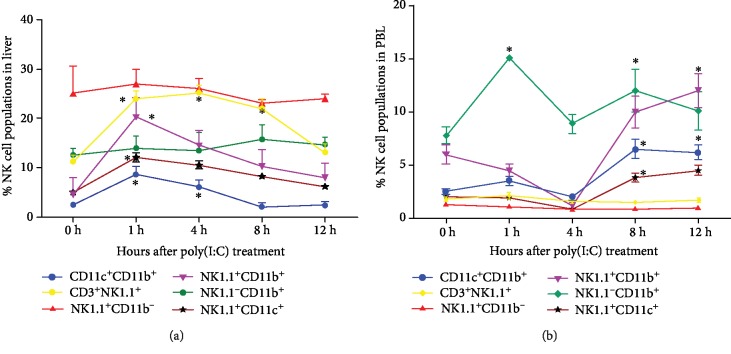
Kinetics of NK subsets after the treatment with poly(I:C) in the PBL and liver. Naïve C57BL/6 mice (*n* = 6 mice/group) were treated with 200 *μ*g poly(I:C) in 200 *μ*l and then sacrificed after 1, 4, 8, and 12 hours after injection. Single-cell suspensions were prepared of the PBL and liver and stained with antibodies against NK1.1, CD3, CD11b, and CD11c and analyzed by 3-color flow cytometry. The kinetics of NK1.1^+^CD11b^+^, NK1.1^+^CD11c^+^, NK1.1^+^CD11b^−^, NK1.1^−^CD11b^+^, CD3^+^NK1.1^+^, and CD11c^+^CD11b^+^ are shown in the (a) PBL and (b) liver. The data represents the mean ± SD at each time point of each group. ^∗^*P* < 0.05, ^∗∗^*P* < 0.01.

Next, at each of the time points, the kinetics associated with the alterations in the NK cell subsets were analyzed in both the liver and the PBL. To achieve this, a single treatment of poly(I:C) (200 *μ*g/mouse) was injected into the mice. The mice were then bled and euthanised at the four time points following the treatment (1 hr, 4 hr, 8 hr, and 12 hr). Control mice were injected interperitoneally with PBS (200 *μ*l), and these were taken as the control time point of 0 hr. At the 1 hr time point, there were no significant alterations in the PBL compartment for the relative number of the NK1.1^+^CD11b^+^ subpopulation of cells following the treatment. However, at the 4 hr time point, the numbers of this subpopulation were lower. At the 8 hr and 12 hr time points, their numbers were raised quickly (Figure 3(a)). Contrastingly, the liver showed a rapid rise in this NK cell subpopulation (NK1.1^+^CD11b^+^) at the 1 hr time point. At the three subsequent time points, however, there was an evident slow decrease in their levels until it recovered to the level of the control (Figure 3(b)).

The NK1.1^+^CD11b^−^ cell subpopulation was not influenced significantly following the poly(I:C) treatment in the PBL, but their level in the liver was increased at the 1 hr time point, then it showed a slow recovery at the three subsequent time points sitting at a higher level than the control at the 12 hr time point (Figure 3(b)). In PBL, the level of the NK1.1^−^CD11c^+^ cell subpopulation remained unaffected at 1 hr, then increased at 4 hr with a further increase at both 8 hr and 12 hr (Figure 3(a)). This NK cell subset (NK1.1^−^CD11c^+^), however, showed a great increase in level in the liver at 1 hr, followed by a slow recovery to their control point through the next three time points (Figure 3(b)). The NKT (NK1.1^+^CD3^+^) cell subpopulation was not significantly impacted by the poly(I:C) treatment in either the PBL (Figure 3(a)) or the liver (Figure 3(b)) at all four time points.

We also analyzed the numbers of monocyte and macrophage (NK1.1^−^CD11b^+^) cells and the number of myeloid DCs (CD11b^+^CD11c^+^, myeloid dendritic cells) to determine whether the other cell populations were also impacted following the treatment with poly(I:C). The monocytes and macrophages were examined in the liver and blood and the myeloid DCs in the liver and PBL. In the blood, the monocyte numbers were raised at all four time points, but being the highest at the 1 hr time point (Figure 3(a)). Figure 3(b) illustrates the number of macrophages (NK1.1^−^CD11b^+^) in the liver which were raised rapidly at the 1 hr time point and then showed a gradual recovery to the control level throughout the remaining three time points. DC phenotypes (NK1.1^−^CD11b^+^CD11c^+^) were raised in the liver and the blood, but the kinetics of the alterations varied. In the liver, they were raised at the 1 hr time point, and then they recovered to control levels through the next three time points (Figure 3(b)). In the blood, the number of these levels was raised initially at the 8 hr time point and then subsequently at the 12 hr time point (Figure 3(a)).

### 3.2. NK Cells with NK1.1, CD11b, CD11c, and B220 Expression Are Induced by Poly(I:C)

**Figure 4:**
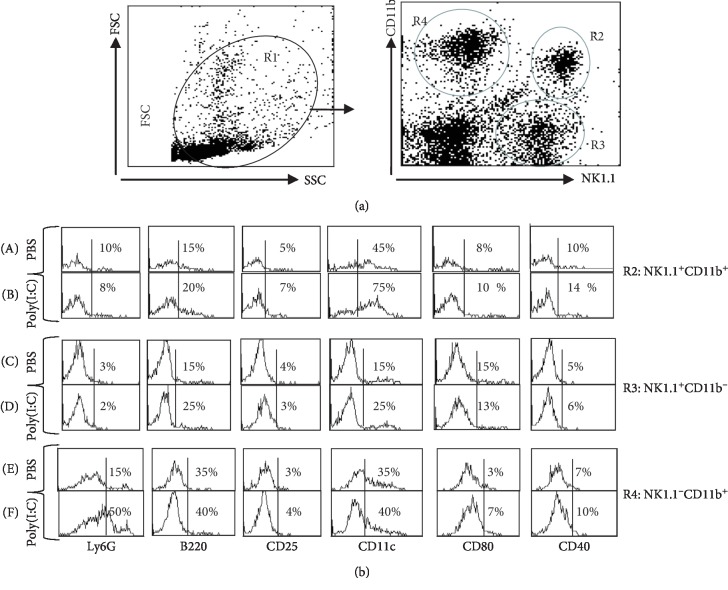
The phenotypic analysis of NK cell subsets in the liver after the treatment with poly(I:C). Naïve C57B/6 mice (*n* = 6 mice/group) were treated with 200 *μ*g poly(I:C) and then sacrificed after 4 hours postinjection. Single-cell suspensions were prepared of liver and stained with antibodies against NK1.1, CD11b, Ly6G, B220, CD25, CD11c, CD80, and CD40 and analyzed by 3-color flow cytometry. The phenotype of NK cell subsets is shown in the liver of control and treated mice with poly(I:C).

As illustrated in Figure 4, it was clear in the liver that poly(I:C) instigated alterations in the NK1.1^+^CD11b^+^ (R2) and NK1.1^−^CD11b^+^ (R3) cell subsets in the liver. Consequently, we sought to examine at 1 hr following the treatment the cell population phenotypes. Furthermore, the NK1.1^+^CD11b^−^ (R4) phenotype was also analyzed. To this end, R2, R3, and R4 were gated from the remaining total leukocyte population (R1) (Figure 4(a)).

The NK1.1^+^CD11b^+^ (R2) cell population in the control mice had low levels of expression of Ly6G and of B220. The cells with NK1.1^+^CD11b^+^ phenotype expressed appreciated levels of CD11c molecule, whereas CD40 and CD80 expressions were almost absent (upper panel of Figure 4(b)). Following the treatment with poly(I:C), however, the NK1.1^+^CD11b^+^ cell subset showed higher expression levels of CD11c and B220 and lower CD80, CD40, and Ly6G levels when compared to PBS-treated mice. Given that CD11c expression was higher on NK1.1^+^CD11b^+^ (R2, Figure 4) cell population than on NK1.1^+^CD11b^−^ (R3, Figure 4) population, we suggest that poly(I:C) seems to increase the relative numbers of NK1.1^+^CD11b^+^CD11c^+^ more than those of NK1.1^+^CD11b^−^CD11c^+^ subpopulations.

Next, in the control mice, the NK1.1^+^CD11b^−^ (R3) subpopulation expressed low levels of B220, CD11c, and Ly6G following the poly(I:C) treatment as illustrated in Figure 4(b). In keeping with the R2 population, the number of NK1.1^+^CD11b^−^ expressing CD25, CD80, and CD40 was also low, indicating that the poly(I:C) treatment did not activate these cell populations as illustrated in Figure 4(b) (C, D).

Analyzing the expression levels of R4 in the liver leukocytes, that of the NK1.1^−^CD11b^+^ phenotype, we find B220 and Ly6G to be expressed at lower levels in the control mice with appreciated levels of expression of CD11c as illustrated in Figure 4(b), B, D, and F. The number of NK1.1^−^CD11b^+^ cells in the liver expressing CD40, CD80, and CD25 was not impacted by the poly(I:C) treatment as they sowed low levels of expression (Figure 4(b), B, D, and F).

### 3.3. CD11b Was Needed for the Impact of Poly(I:C) on NK1.1^+^CD11b^+^ Cells

From the above findings, it is clear that the treatment with poly(I:C) led to a rise in the relative numbers of both NK1.1^−^CD11b^+^ and NK1.1^+^CD11b^+^in the liver. Thus, this indicates that for poly(I:C) to have an impact, CD11b and NK1.1 molecules might be required to facilitate the effect.

**Figure 5:**
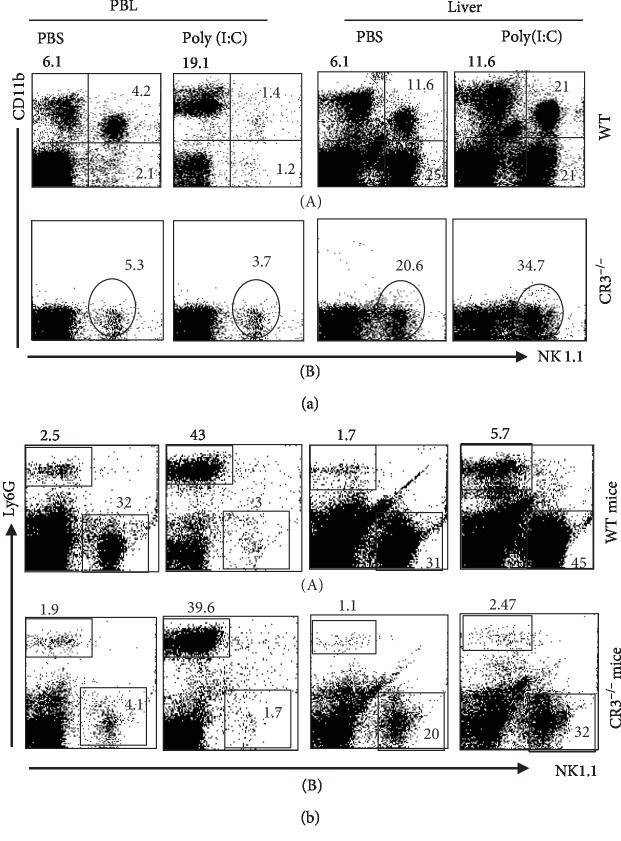
Influence of CR3 deficiency on the effect of poly(I:C) on the frequency of NK cells. Naïve C57BL/6 mice and CR3^−/−^ mice (*n* = 6 mice/group) were treated with 200 *μ*g poly(I:C) and then sacrificed 4 hours postinjection. Single-cell suspensions were prepared of PBL and liver and stained with antibodies against NK1.1, CD11b, and Ly6G and analyzed by 3-color flow cytometry. (a) The frequency of NK cell (NK1.1^+^CD11b^+^) and NK cell (NK1.1^+^) populations. (b) The frequency of NK1.1^−^ Ly6G^+^ and NK1.1^+^ Ly6G^−^ populations.

To help establish the role of CD11b, we contrasted between the impact of poly(I:C) on the relative number of cells of NK1.1^+^CD11b^+^ in the liver and PBL in CR3^−/−^ (CD11b) knockout mice and wild-type mice. In keeping with the findings shown in Figure 1(a), the outcome in the blood of wild-type mice showed that poly(I:C) led to a reduction in the NK cell population NK1.1^+^CD11b^−^ from 4.2% to 1.4% (Figure 5) linked to a rise in the level of NK1.1^−^CD11b^+^ cells from 6.1% to 19.1% (Figure 5(a), A). These effects were not evident in the knockout mice (CR3^−/−^) since these mice do not express CD11b receptor as shown in Figure 5(a), B. Of note, poly(I:C) had no evident effect on the numbers of NK cells in the blood with NK1.1^+^CD11b^−^ phenotype in the wild type and in CR3^−/−^ mice (Figure 5(a)).

Contrastingly, poly(I:C) still had an impact on the NK cells of the liver even when CD11b was not present as the outcome indicated a rise in the relative numbers of NK cells with NK1.1^+^CD11b^−^ phenotype as shown in Figures 5(a), B, and 5(b), B. These data indicate that in the absence of CD11b molecule, poly(I:C) can still increase the remained numbers of NK cells in the liver but not in the blood.

**Figure 6:**
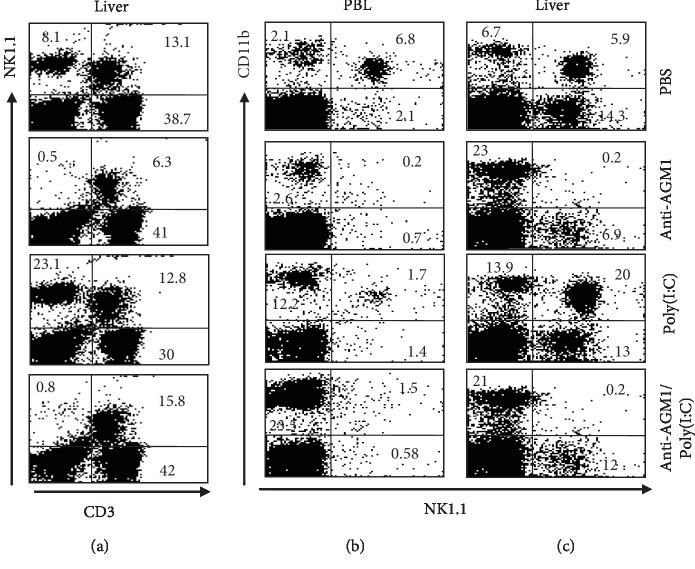
Influence of NK cell deficiency on the effect of poly(I:C) on the frequency of NK cells. Naïve C57BL/6 mice and NK-depleted mice (*n* = 6 mice/group) were treated with 200 *μ*g poly(I:C) and then sacrificed 4 hours post injection. Single-cell suspensions were prepared of PBL and liver and stained with antibodies against NK1.1, CD11b, and CD3 and analyzed by 3-color flow cytometry. (a) The frequencies of NKT cells (CD3^+^NK1.1^+^) cells and CD3^−^NK1.1^+^ cells. (b, c) The frequencies of NK1.1^−^CD11b^+^ and NK1.1^+^CD11b^+^ cells.

As Ly6G was found to be coexpressed in CD11b-expressing cells, we examined the expression of Ly6G in CR3^−/−^ knockout mice following the treatment with poly(I:C). Only a very small number of cells coexpressed Ly6G and NK1.1 in the liver and the blood (Figure 5(b)). Following the treatment with poly(I:C), the numbers of Ly6G^+^NK1.1^−^ cells were increased from 2.5% to 43% in the blood of wild type and from 1.9% to 36.9% in CR3^−/−^ knockout mice (Figure 6(b)). In the liver, poly(I:C) increased the numbers of these (Ly6G^+^NK1.1^−^) cells from 1.7% to 5.7% in the wild-type mice and from 1.1% to 2.7% in the knockout mice. The numbers of NK1.1^+^Ly6G^−^ were lowered in the PBL of both wild type (from 32% to 3%) and in knockout (from 4.1% to 1.7%) mice while they were increased in the liver of wild type (31% to 45%) and in knockout (from 20% to 32%) mice as demonstrated in Figure 5(b), B. Taken these data together, it seems that poly(I:C) increases and decreases the numbers of Ly6G^+^ cells and NK1.1^+^, respectively, in the blood of CR3^−/−^ knockout mice while it increases the numbers of both cells in the liver of both wild-type and CR3^−/−^ knockout mice.

Overall, these findings indicate that the role of CD11b in the effects of poly(I:C) on NK cells varies in the blood and liver. In the blood, the number of NK cells was lowered, and this correlated with a rise in their levels in the liver.

The absence of NK1.1^+^CD11b^+^ cells maximizes the effects of poly(I:C) on NKT in the blood and CD11b^+^NK1.1^−^ cells and CD11b^−^NK1.1^+^ cells in the liver.

We proceeded to examine the significance of NK1.1 in facilitating the impact of poly(I:C). In both the blood and the liver, NK1.1^+^CD3^−^ and NK1.1^+^CD11b^+^ cell populations are depleted in the blood by anti-AGM1, which also depletes NKT (NK1.1^+^CD3^+^) and NK1.1^+^CD11b^−^ cells partially (Figure 6). Treatment with anti-AGM1, however, increased the relative numbers of NK1.1^−^CD11b^+^ cell population in the liver (from 6.7% to 23%), but not in the blood (Figure 6).

Thus, using anti-AGM1, we examined the impact and determined that the treatment with poly(I:C) following injection of mice with anti-AGM1 led to the normalization of the reduced level of NKT cells in the PBL as illustrated in Figure 6(a). In keeping with Figure 1(a), 4 hr following the poly(I:C) treatment of wild-type mice resulted in a reduction in the levels of NK1.1^+^CD11b^+^ and increased in the levels of NK1.1^−^CD11b^+^ from a blood level of 2.1 to 12.2 as illustrated in Figure 6(b). When mice were treated with poly(I:C) following the treatment with anti-AGM1, here, the PBL showed further rise in the relative numbers of NK1.1^−^CD11b^+^ cell population (from 12.2% to 23.5%), while the lowered numbers of NK1.1^+^CD11b^+^ cell population were not affected (Figure 6(b)). These data indicate that in the absence of NK1.1^+^CD11b^+^ cells and partial absence of CD3^+^NK1.1^+^ (NKT cells), poly(I:C) can normalize the partial decrease in the numbers of NKT cells and increase the numbers of CD11b^+^NK1.1^−^ cell population in the blood.

The liver, on the other hand, gave the expected results with the treatment of wild-type mice with poly(I:C) showing a raise in NK1.1^−^CD11b^+^ relative numbers from 6.7% to 13.9%. Moreover, NK1.1^+^CD11b^+^ relative numbers also increased from 5.9% to 20%. The treatment of mice with anti-AGM1 Ab depleted the relative numbers of NK1.1^+^CD11b^+^ cell population and partially decreased the relative numbers of NK1.1^+^CD11b^−^ cell population (from 14.3% to 6.9%) in the liver while increased those of NK1.1^−^CD11b^+^ cell population (from 6.7% to 23%) (Figure 6(c)). Contrastingly, the treatment of the wild-type mice with anti-AGM1 first followed by poly(I:C) increased the numbers of NK1.1^+^CD11b^−^ cell population to 12% as compared to mice treated with anti-AGM1 Ab (6.7%) as illustrated in Figure 6(c). Likewise, there was an agreement between the absolute numbers of these subsets following poly(I:C) injection when compared to the relative numbers (data not presented). Thus, these findings demonstrated that when NK1.1^+^CD11b^+^ is not present in the liver, poly(I:C) has an impact on CD11b^−^NK1.1^+^, but not on CD11b^−^NK1.1^+^ cell subsets.

Taken together these findings on the effects of poly(I:C) on the AGM1-treated mice, it can be demonstrated that depletion of NK1.1^+^CD11b^+^ cells in the blood and liver by anti-AGM1 Ab makes poly(I:C) to increase the numbers of CD3^+^NK1.1^+^ (NK T cells) in the blood and the numbers of CD11b^+^NK1.1^−^ cell populations in the blood and liver.

### 3.4. The Impact of Poly(I:C) on NK Cells Is In Part Reliant on TLR3

**Figure 7:**
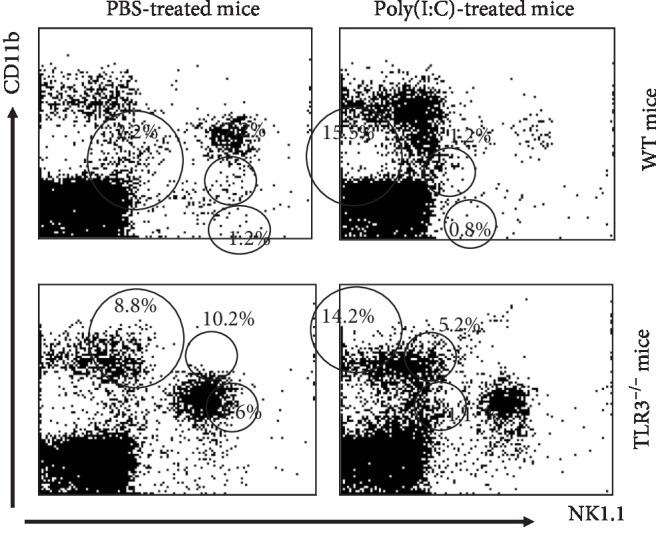
Influence of TLR3 deficiency on the effect of poly(I:C) on the frequency of NK cells. Naïve C57BL/6 mice and TLR3^−/−^ mice (*n* = 4 mice/group) were treated with 200 *μ*g poly(I:C) and then sacrificed 4 hours postinjection. Single-cell suspensions were prepared of liver and stained with antibodies against NK1.1 and CD11b and then analyzed by 2-color flow cytometry. The frequencies of NK1.1^−^CD11b^+^ and NK1.1^+^CD11b^+^ cells are shown.

The initiation of the target cells by poly(I:C) is partly, but not fully, due to its association with TLR3, its cognate receptor. To establish how significant TLR3 is for the impact of poly(I:C) on NK cells, the part of TLR3 was examined by establishing the NK cell numbers in TLR^−/−^ and wild-type mice following the poly(I:C) treatment. The relative numbers of cells of the NK1.1^+^CD11b^+^ subset showed a 2-fold decrease in the blood, which was compared to a larger decrease (7 fold) in the wild-type mice following the treatment with poly(I:C) as illustrated in Figure 7.

The poly(I:C) treatment of mice from the wild-type sample had a 7-fold rise in the relative numbers of CD11b^+^NK.1.1^+^ cells, while the knockout mice only showed a 2-fold increase in numbers. This demonstrated a partial role played by TL3 in the facilitating of the effect of poly(I:C). This partial part was expected as poly(I:C) can also activate target cells through a protein kinase R (PKR) [40].

## 4. Discussion

Several studies, including our earlier studies, indicated that the administration of poly(I:C), a TLR3 agonist *in vivo*, into naïve mice led to the quick rise in NK cell numbers primarily in the liver and that this was linked with greater antitumour effectiveness and antigen-specific reactions [18, 21, 36, 41]. In this research study, we examined the phenotypic characteristics of the various NK cell subpopulations that included NK1.1^+^CD11b^+^, NK1.1^+^CD11c^+^, NK1.1^+^CD11b^−^, and NK1.1^+^CD3^+^. This was performed at various time points and primarily in the liver and the PBL. Three significant observations were made. First, NK cell numbers in the peripheral blood decreased following the poly(I:C) treatment at the 4 hr time point. Second, following the poly(I:C) treatment, numbers of the NK1.1^+^CD11b^+^ cell population increased significantly in the liver. Third, NK cells expressing high levels of NK1.1, CD11b, and CD11c were increased while those expressing Ly6G and B220 were decreased following the treatment with poly(I:C). Thus, these findings indicate to the rise in the number of NK cells in the liver that are of a particular phenotype as a result of the poly(I:C) treatment.

The populations of NK cells in mice and in humans are formed of several subpopulations. Human NK cells can be split into those with high cytotoxicity and which are present in the peripheral blood with the CD56^dim^ phenotype and others of low cytotoxicity that are found in lymphoid organs with the phenotype CD56^bright^ [42, 43]. In addition to these, NK cells in mice are split into NK1.1^+^CD11b^low^ and NK1.1^+^CD11b^high^ [16]. Other research has differentiated between NK cell subpopulations using their surface density of CD11b and CD27, and this resulted in the following four subpopulations: CD11b^low^CD27^low^, CD11b^low^CD27^high^, CD11b^high^CD27^high^, and CD11b^high^CD27^low^ [44]. In this study and in the previous ones, we targeted NK cells that expressed either high or low levels of CD11b as we had determined that most NK cells were accounted for by these subpopulations (data not presented).

In keeping with other literature [36, 41], our findings demonstrated that the poly(I:C) treatment resulted in a 2-fold increase in the relative numbers of NK cells with the NK1.1^+^CD11b^+^ and NK1.1^+^CD11c^+^ phenotypes in the liver. This occurred quickly only 1 hr following the treatment and was also noted at the 4 hr time point, although the latter was not as significant. Recovery back to control, or pretreatment levels, was noted after the 12 hr time point. This was not the case, however, in peripheral blood where the treatment with poly(I:C) instigated a gradual reduction in NK cell subpopulations with phenotypes of NK1.1^+^CD11b^+^ and NK1.1^+^CD11c^+^ at the time of 4 hr following the treatment. Subsequently, a large rise in the numbers of these two subpopulations was noted at 24 hr following the treatment. Of note was the lack of significant changes in the numbers of NK1.1^+^CD3^+^ NKT cells in the blood and the liver. Moreover, in addition to the alterations seen with the relative numbers of NK1.1^+^CD11b^+^ in the blood and liver following the poly(I:C) treatment, a rise in the number of NK cells of the phenotypes of NK1.1^−^CD11b^+^ and CD11b^+^CD11c^+^ and NK1.1^+^CD11b^−^ was also noted in the liver but were not observed in the blood. Overall, these findings indicate that poly(I:C) initiates changes in the distribution of cells NK cells with NK1.1^+^CD11b^+^ cells by initiating a quick rise in the numbers of these subpopulations in the liver and that this correlates with an initial immediate decrease in the blood followed by a rapid rise.

These differences in the impact of poly(I:C) on the NK cell subsets in the liver and the blood could be accounted for by a difference in the impact of poly(I:C) as dependent on the compartment being affected. This difference between compartments would be the result of NK1.1^+^CD11b^+^ initially being trafficked from the blood to the liver and then subsequently from the liver to the blood at the latter time points. This suggestion is in line with earlier research that demonstrated how the poly(I:C) treatment initiated accumulation of active NK cells in the liver through the inducing of these cells to migrate from the spleen through to the liver [41]. These findings shed a light on the antitumour impact of poly(I:C) on metastatic tumours given that such tumours secrete interferon as we have previously shown [18, 21, 26, 39] and that NK cells have a part in immune surveillance against cancerous cells [45] and that they are able to eradicate cancers from the liver [8, 33]. These findings also contribute to future research in this area in further examining the impact of the poly(I:C) treatment against liver tumours when taken systemically or via hepatic portal injection.

In keeping with the idea that inducing of TLR3 in NK cells can enhance NK cell-facilitated antitumour functions, *in vitro* research in humans showed that TLR4L-activated NK cells are capable of eliminating autologous bile ducts in cases of primary biliary cirrhosis when IFN-*α* that is synthesised by TLR3L-stimulated monocytes is present. Moreover, there are large rises in the cytotoxic function of hepatic NK cells in patients of primary biliary cirrhosis compared to control NK cells. This only occurs, however, when these NK cells were prepared after ligation of both TLR3L-stimulated liver mononuclear cells [34]. Additionally, the activation of TLR3 following the poly(I:C) treatment resulted in a rise in cell death in the TLR3^+^ SNU182 hepatocellular carcinoma cell line while also enhancing stimulation of NK cells and their cytotoxicity by 4-fold *in vitro* [35]. Moreover, the performance of *in vitro* research on cancer cell lines and on NK cells demonstrated that the power of NK cytotoxicity was enhanced greatly following the treatment with poly(I:C) in Tu167 cells, a head and neck squamous cell carcinoma cell line. This was also the case against the IFN-*ɣ* expression in the NK92 cell line, human CD56^+^CD3^−^ primary NK cells, and the erythroleukemia K562 cells associated with upregulated TNF-*α*. Intratumoural chemokine expression was raised *in vivo*, together with tumour infiltration, NK cell activation, and NK cell and T cell proliferation, and tumour infiltration following the poly(I:C) treatment. This also correlated with reduced levels of proliferation of tumour parenchyma cells. This research study also demonstrated that there was an association between the expression of TLR3 in the samples of tumour patients together with T cell and NK cell tumour infiltration, NK cell activation and that it was indirectly associated with the viability of tumour parenchyma cells and with longer patient survival [35]. Thus, these research studies demonstrated the vital role played by TLR3 in the modulation of progression of HCC, thereby, making it a potential target for new immunotherapeutic approaches that are based on antitumour cytotoxicity of poly(I:C)-stimulated NK cells and of macrophages [46].

To comprehend the exact NK cell phenotype that is impacted by poly(I:C), we conducted an in-depth phenotypic analysis on liver NK cells following the poly(I:C) treatment on the various NK cell subpopulations. These analyses indicated that the poly(I:C) treatment initiated rises in the NK cell numbers that express high levels of NK1.1, CD11b, and CD11c molecules and low levels of Ly6G and B220 molecules. It was also noted that this phenotype lacked CD40 and CD80, two costimulatory molecules. Overall, these findings demonstrate that NK cell subsets in the liver expressing the CD11c and CD11b myeloid markers are stimulated by TRL3L poly(I:C).

Next, the part played by TLR3 was examined together with the shared interaction between NK cell expressing CR3 and those expressing CD11b. Firstly, these findings demonstrated the impact of poly(I:C) on the rise in NK cell numbers. They demonstrated that it was partially facilitated by TLR3 presence. This was expected as both protein kinase R (PKR) and TLR3 can both induce target cells following their stimulation by poly(I:C) [47, 48]. We then sought to determine whether the impact of poly(I:C) on NK cells is facilitated by CD11b receptors being present. We found that the treatment with anti-AGM1 antibody resulted in elimination of NK1.1^+^CD11b^+^ cells and partial elimination of NK1.1^+^CD3^+^ cells and NK1.1^+^CD11^−^ cells coincided with a 4-fold rise in macrophages (NK1.1^−^CD11b^+^) numbers in the liver. Injection of poly(I:C) into these anti-AGM1-treated mice resulted in recovery of NK1.1^+^CD3^+^ and NK1.1^+^CD11b^−^ cells to their control levels. Interestingly, lack of CD11b (CR3) expression (in CR3^−/−^ mice) did not interfere with the effect of poly(I:C) to increase the numbers of the remained NK cells (NK1.1^+^CD11b^−^ and NK1.1^+^CD3^+^ populations).

Earlier studies had demonstrated how in cases of CD11b-deficient NK cells, following the treatment with or without poly(I:C), that the *β*_2_ integrin CD11b attenuate poly(I:C)-induced hepatitis resulted in greater power of cytotoxicity and a greater level of IFN-*ɣ* and granzyme B released. Moreover, *in vivo* adoptive transfer of CD11b-deficient NK cells into NK cell depleted mice showed that the function of CD11b-mediated suppression of NK cells is the cause of the attenuation of poly(I:C)-induced acute hepatitis by CD11b [49]. Nevertheless, it is clear that the part played by CR3 in the adjuvant impact of poly(I:C) should be studied in future research.

We do believe that the effect of poly(I:C) could be mediated partly by both its effect on CD11b^+^ on NK cells as well as by an event mediated through CD11b signalling. The latter suggestion is based on the report by Hui Zhou et al. [50] who showed CD11b/CD18 (Mac-1) as a novel surface receptor for extracellular double-stranded RNA (dsRNA, e.g., poly(I:C) to mediate cellular inflammatory response. This dsRNA activates CD11b to enhance TLR3-dependent signalling pathway, which trigger CD11b-dependent inflammatory response. Similarly, the adjuvant effect of exogenous administration of poly(I:C), as in our study, might be mediated by similar events.

In one of our recent studies, we used the clinical grade (Hiltonol®) of poly(I:C) in a clinical trial of pancreatic cancer to enhance vaccination with DCs loaded with tumour peptides [51]. Hiltonol® was used for the purpose to activate DCs *in vitro* before loading the peptides as well as *in vivo* by its administration along with DC vaccine at the vaccination site. Under this setting, we suggest that systemic administration of poly(I:C) induces increases in the numbers of NK cell subpopulations in the liver and stimulate them to optimize antigen-specific vaccination. A modified form of poly(I:C) termed ARNAX consists of DNA-capped dsRNA that specifies the endosomal target for TLR3 in DCs has also been developed and tested for immunotherapeutic approaches in preclinical studies using mouse models [52]. This modified form of poly(I:C) has been proposed to be safe for administration in elderly patients with cancer receiving immunotherapy.

To summarise, poly(I:C), the TLR3-agonist, instigates an NK cell subset that is found to express CD11b and CD11c, two myeloid markers. The increase in the numbers of this NK cell population might be clinically significant. For example, the cells can be targeted to stimulate antitumour antibody-dependent cytotoxicity (ADCC) by a bispecific antibody that is able to link with tumour-associated antigen on tumour cells and to NK cells through their FCɤRI region.

Overall, we suggest that poly(I:C) can be used as adjuvant therapy against hepatitis viruses or liver cancer to enhance the resultant immunity.

## Data Availability

The data used to support the findings of this study are available from the corresponding author upon request.
